# Clinical Analysis of the Diagnostic Accuracy and Time of Execution of a Transillumination Caries Detection Method Compared to Bitewing Radiographs

**DOI:** 10.3390/jcm10204780

**Published:** 2021-10-19

**Authors:** Gian Andrea Pelliccioni, Maria Rosaria Antonella Gatto, Silvia Bolognesi, Daniele Dal Fiume, Maicon Sebold, Lorenzo Breschi

**Affiliations:** 1 Department of Biomedical and Neuromotor Sciences, Alma Mater Studiorum, University of Bologna, Via San Vitale, 59, 40125 Bologna, Italy; gian.pelliccioni@unibo.it (G.A.P.); bolognesisilgin@gmail.com (S.B.); lorenzo.breschi@unibo.it (L.B.); 2 Dal Fiume Private Practice, Via G. Leopardi, 101, 40026 Imola, Italy; daniele.dalfiume70@gmail.com; 3 Department of Restorative Dentisty, Operative Dentistry Division, Piracicaba Dental School, University of Campinas, Avenida Limeira, 901, Piracicaba 13414-903, Brazil; maicon_sebold@hotmail.com

**Keywords:** fiber optic technology, transillumination, radiography, bitewing, dental caries, diagnosis, oral, ROC curve

## Abstract

Purposes: this clinical study evaluated the accuracy and execution time of a digital imaging fiber-optic transillumination (DIFOTI) technique for the detection of approximal caries in posterior teeth compared to intra-oral examination associated with bitewing radiographs. Methods: one hundred patients were selected and submitted to clinical inspection and bitewing radiographs. The outcomes of this process were converted into scores, according to the International Caries Detection and Assessment System (ICDAS): 0—sound tooth; 1, 2, and 3—carious lesion confined within enamel; 4, 5, and 6—dentin carious lesion. Subsequently, an independent investigator acquired digital images of the same teeth using a DIFOTI device (DIAGNOcam, Kavo Dental), which were also converted into ICDAS scores. The time required for executing diagnostic procedures was measured. The clinical sensitivity and specificity of DIFOTI were analyzed by receiver operating characteristic (ROC) curves. The time necessary to perform the diagnostic methods was evaluated by Mann–Whitney U (alfa = 0.05). Results: the overall test accuracy for the DIFOTI-based device ranged from 0.717 to 0.815 (area under the ROC curve) with *p* < 0.0001 for all ICDAS scores. Bitewing radiographs took almost twice the time required by DIFOTI (*p* < 0.001). Conclusions: the DIFOTI-based device DIAGNOcam provided accurate detection of approximal caries in posterior teeth, even at early stages. The technique employed for transillumination caries diagnosis by the same device took less time than conventional bitewing radiographs. Clinical Relevance: transillumination devices, such as DIAGNOcam, can be accurately used for caries diagnosis in approximal surfaces of posterior teeth, demanding less clinical time and without radiation-related risks.

## 1. Introduction

Diagnosis of carious lesions can be performed by means of different methods, including visual/tactile inspection by a professional, followed by bitewing and/or periapical radiographs, as well as transillumination techniques [1]. Although lesions in anterior teeth or in the occlusal surface of posterior teeth can be easily identified by clinical inspection, this procedure fails to detect incipient lesions in approximal surfaces. Therefore, supporting diagnostic techniques, such as radiographs and transillumination [2], are usually applied with the aim of accurately analyzing approximal surfaces, where peculiar anatomic aspects tend to mask the presence of carious lesions. However, bitewing radiographs are known to present great variability (6–70%) regarding in vivo sensitivity due to its inability to detect interproximal carious lesions at their early stages [3].

Hence, transillumination diagnostic methods have been developed, and a near-infrared fluoroscopic device based on digital imaging fiber-optic transillumination (DIFOTI) has been released on the market [4]. This technology applies a transmitted inspection light that is detected by a charge-coupled device (CCD) camera using laser light. In other words, near-infrared rays (approximately 780 nm) are emitted by the device through the tooth and surrounding anatomic structures in order to obtain images from occlusal surfaces [5,6]. An in vitro investigation demonstrated that DIFOTI was more effective and accurate to detect approximal enamel carious lesions at their onset compared to film and digital radiographs [7]. Moreover, another in vitro study comprising image and histologic analyses of premolars and molars, without occlusal restorations or approximal calculus, reported that a near-infrared laser transillumination (NIR-LTI) technique showed higher sensitivity, accuracy, and area compared to conventional radiographs for incipient caries detection [8].

Additionally, a clinical study by Söchtig et al. (2014) compared a DIFOTI-based diagnostic device with traditional bitewing radiographs, showing 95.3% of the near-infrared light images agreed with the bitewing radiographs for dentin caries detection [6]. Thus, other clinical studies have been performed in an attempt to validate the transillumination diagnostic approach, showing it has potential to be a useful supporting technique in caries control [9,10,11]. Even so, there is a lack of evidence in the literature to suggest the replacement of traditional radiographic exams by transillumination devices, and the limitations, indications, and usefulness of this alternative diagnostic method still need to be explored.

The use of a DIFOTI-based device could lead to faster and more straightforward image acquisition for caries detection. This would be advantageous in clinical practice to save time during patient triage, which could even be performed by an experienced and well-trained dental hygienist before consultation with the responsible dentist. Thus, in order to evolve current knowledge regarding DIFOTI technology and help validate this diagnostic method, the objective of the present clinical study was to evaluate and describe the performance of the DIFOTI-based device DIAGNOcam (KaVo Dental, Genova, GE, Italy) for the detection of approximal carious lesions in posterior teeth compared to intra-oral examination coupled with bitewing radiographs. The accuracy of a transillumination technique for approximal caries diagnosis, measured by receiver operating characteristic (ROC) curves, was determined in comparison to bitewing radiographs coupled with clinical inspection. The time required to acquire radiographs or digital images by the diagnostic methods was also measured and compared. The study hypotheses were that (1) the use of a transillumination device would be as accurate as traditional diagnostic methods (visual examination coupled with phosphor plate-acquired bitewing radiographs) for the detection of posterior approximal carious lesions and that (2) the DIFOTI-based device would require significantly less time to perform the examination process.

## 2. Material and Methods

### 2.1. Patient Selection and Data Collection

One hundred patients were selected from the Department of Restorative Dentistry of the University of Bologna and from a private practice located in Imola (BO, Italy), after the research protocol was explained to them and they signed an informed consent form. This study was approved by the Ethical Committee Bologna-Imola (18106–18130\2018), and it was conducted from the 15 January until 15 March 2019. Eligibility criteria were age (12–35 years old) and the American Society of Anesthesiologists Classification ASA I (normal healthy patients). Conversely, patients with total dentures or fixed orthodontic appliances were excluded from the study protocol.

The sample size was estimated from the calculation of the standard error of the mean difference between the results of the two methods at a 95% confidence interval, according to the following formula by Bland & Altman (1986) [12]:

$$d=\sqrt{\left(\frac{3s^{2}}{n}\right)}$$

where *d* is the standard error of the mean difference, *s* is the standard deviation of the difference, and *n* is the sample size. The number of 100 research subjects was considered adequate, given the small *d* value of ±0.34 s at a confidence interval of 95%.

Each patient was coded by a number to protect their identity. Two experienced practitioners (G. A. P. and D. D. F.) carried out clinical examination after previous calibration according to the International Caries Detection and Assessment System (ICDAS) score [13]. All posterior teeth (upper and lower pre-molars and molars) of each patient were included in the exam, except for third molars, which were not considered in the investigation. Teeth were examined regardless of the presence of occlusal and/or approximal restorations. Clinical exams were performed by an intra-oral mirror and a dental explorer on all teeth of each patient, after removing supragengival calculus and cleaning the teeth, followed by digital bitewing radiographs (CS 7600 scanner, Carestream Dental, LLC, Atlanta, GA, USA). All diagnostic data collected during anamnesis and clinical/radiographic exams were interpreted and converted into ICDAS scores, being then registered on electronic forms. Afterwards, a trained dental hygienist (S.B.), blinded to the results of the initial examination, reassessed the patients with a DIFOTI-based device (DIAGNOcam). Having a trained, experienced dental hygienist who could acquire the DIFOTI images and even pre-evaluate them before the dentist sees the patient could be very useful in daily practice to save time and optimize patient care. All procedures performed by the dental hygienist were supervised by a trained dentist. Transillumination images were obtained by placing the device parallel to the occlusal surfaces of the posterior teeth previously investigated by clinical examination and bitewing radiographs. DIFOTI data was also converted into ICDAS scores and added to the electronic forms. Moreover, the time necessary to execute either bitewing radiographs or DIFOTI images was registered for comparison between the techniques.

### 2.2. Data Interpretation According to ICDAS Score

Both bitewing radiographs and DIFOTI images were assessed by two independent researchers (G.A.P. and S.B.), who consulted a third examiner in case of disagreement (D.D.F.). Radiographs were examined according to O’Mullane criteria [14], comprising mesial and distal surfaces: 0—full thickness of enamel and dentin visible and sound; 1—radiolucency confined to the outer half of the enamel; 2—radiolucency extending as far as the amelodentinal junction (ADJ) but not beyond; 3—radiolucency in enamel and dentin but not involving pulp. Subsequently, the presence or absence of carious lesions recorded by the O’Mullane criteria was fitted into the ICDAS classification as follows: score 0—sound tooth surface with no evidence of caries; scores 1, 2, and 3—carious lesion confined to the enamel tissue; scores 4, 5, and 6—carious lesion with dentin involvement.

Detection of approximal carious lesions by the transillumination device was performed according to a criteria adapted from Lara-Capi et al. (2017) [9], which was later also translated into the ICDAS score by the same researchers mentioned above (G.A.P. and S.B.): 0—sound tooth, and no shadow was detected (ICDAS score 0); 1—a defined yet light and small shadow in enamel was detected due to an incipient lesion (ICDAS score 1); 2—a marked dark shadow with larger dimensions was detected in enamel (ICDAS score 2); 3—a shadow involving enamel and less than 2 mm (up to 1/3) of dentin were detected (ICDAS scores 3 and 4); 4—a shadow involving enamel and more than 2 mm of dentin, eventually reaching the pulp, were detected (ICDAS scores 5 and 6).

The outcome of the association between clinical examination and bitewing radiographs, a procedure commonly performed at the dental clinic for approximal caries diagnosis, was used as the reference standard for the calculation of receiver operator characteristic curves (AUROC). ICDAS scores 1 and 2 were distinguished following the classification described by Gugnani et al. (2011), coupled with radiographic examination: score #1 denoted a clinically visible opacity or white/brown spot on dried enamel surface with a radiolucency in the bitewing radiograph involving only the outer half of enamel; while score #2 showed a visual alteration of the wet enamel surface with a radiolucency reaching as far as the ADJ, but without dentin involvement.

### 2.3. Reliability of Bitewing Radiographs

Interoperator variability was reduced by calibrating the examiners who performed clinical examination and bitewing radiographs. Both operators evaluated the same 10% of the total number of radiographs. In case of disagreement about diagnosis, a third independent examiner was consulted until a consensus was reached. Intraoperator variability, on the other hand, was assessed by Cohen’s kappa, which analyzed the agreement between different measurements by the same examiner one month apart from each other. Cohen’s kappa value was close to one, indicating almost perfect agreement according to Landis and Koch (1997) [15].

### 2.4. Reliability of the Images Captured by Transillumination Technique

The third operator who performed the DIFOTI measurements engaged a sixty-minute training session regarding the transillumination technology and its clinical use prior to taking images from patients. Training was given by one of the authors, who had experience with the specific device used in this study. Then, the operator was asked to diagnose the presence or absence of carious lesions in the images immediately and one month after the training session, using the same criteria at both evaluations. Adequate intraobserver agreement was demonstrated by Cohen’s kappa, which was equal to one.

### 2.5. Recording of the Time Necessary for the Diagnostic Methods Used

The time required for executing bitewing radiographs with photostimulable phosphor plates (Dürr Dental SE, Bietigheim-Bissingen, Germany) was recorded by a digital chronometer (in minutes and seconds) from the moment the protection mantle was placed over the patient until the image was obtained on the computer screen and registered in the chart. The time for acquiring DIFOTI images was recorded from the moment the tip of the device was inserted in the patient’s mouth up until the record of the last picture on the database. Table 1 presents a detailed description of all clinical steps necessary for the acquisition of images by the different diagnostic methods.

### 2.6. Statistical Analyses

All statistical analyses were run by the IBM SPSS software (IBM SPSS Statistics for Windows, Version 25.0, IBM Corp., Armonk, NY, USA). The relationship between clinical sensitivity and specificity of DIFOTI transillumination for each cut-off (ICDAS scores based on the clinical and radiographic evaluation) was analyzed by ROC curves. The number of tooth surfaces identified by each diagnostic method was compared by the Z-test, with a confidence interval of 95%. The time necessary to perform the tested diagnostic methods was evaluated by a non-parametric test (Mann–Whitney U) at a significance level of 95%.

## 3. Results

**Figure 1:**
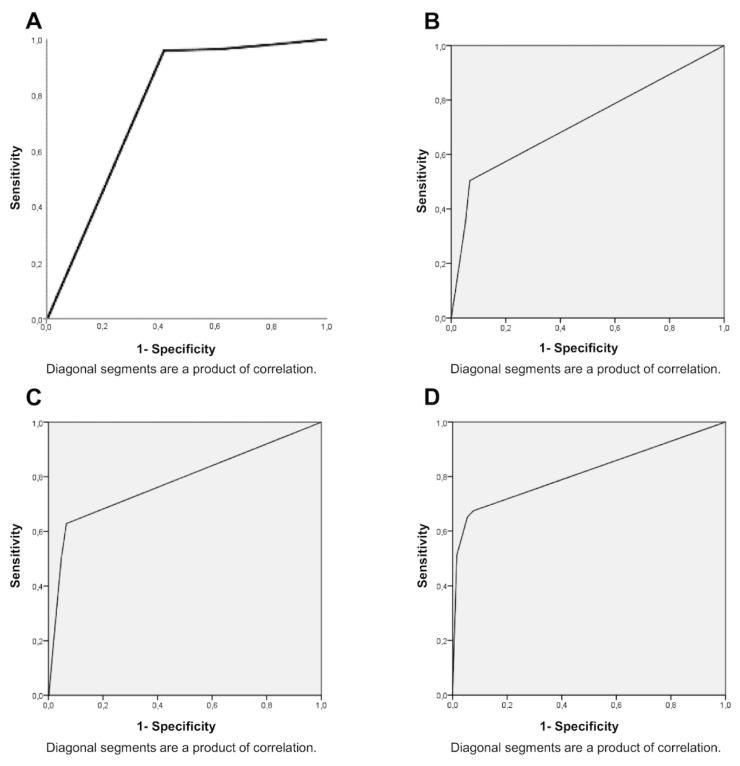
ROC curves (sensitivity x specificity) of the transillumination technique with DIAGNOcamTM when ICDAS scores are equal to 0 (**A**), 1 (**B**), 2 (**C**), or 3 (**D**).

In total, 1760 teeth were evaluated, comprising 3520 surfaces, half of them being mesial and the other half distal. Table 2 presents a detailed description of the number of surfaces classified as each one of the ICDAS scores, according to the diagnostic technique used. This dataset showed a higher number of scores #0 (sound tooth) for the association between bitewing radiographs and clinical examination in comparison to the DIFOTI-based device, suggesting the DIFOTI device led to an overall higher detection of enamel carious lesions. The overall test accuracy for the DIFOTI-based device is reported in Table 3 and Figure 1. Transillumination ranged from 0.717 to 0.815 (area under the ROC curve or AUROC) with *p* < 0.0001 for all ICDAS scores previously set by bitewing radiographs. All graphs presented ascending ROC curves closer to the upper left corner of the plot, representing adequate accuracy for the diagnostic method. Specificity was equal to 96%; sensibility ranked from 33% for ICDAS 1, 52% for ICDAS 2, 69% for ICDAS 3, and 100% for ICDAS 4.

In regard to the clinical time necessary for executing both imaging techniques, bitewing radiographs presented a median of 6 min and 2 s (interquartile range from 3 min and 46 s to 6 min and 25 s), while the DIFOTI-based method showed a median of 3 min and 20 s (interquartile range from 2 min and 59 s to 3 min and 52 s). Bitewing radiographs took almost twice the time required by the DIFOTI device, which was evidenced by a significant difference according to the Mann–Whitney U test (*p* < 0.001). Additionally, bitewing radiographs led to higher variability in their time of execution, as shown by the lower interquartile range of the transillumination technique.

## 4. Discussion

Traditionally, the diagnosis of carious lesions has been based on the association of clinical examination and intra-oral radiographs. Therefore, the International Caries Detection and Assessment System (ICDAS) was proposed in order to standardize the detection, assessment, and management of the carious process at its different stages, considering clinical, radiographic, and histologic information [13,16]. From a clinical point of view, the ICDAS can be defined as a visual score system that indicates the presence of carious lesions from their early onset to clear manifestation thresholds [17,18]. Thence, this score system was selected for the present study.

Bitewing radiographs can be considered an essential supporting tool in dentistry, since they allow the identification of approximal carious lesions in dental areas that cannot be visually inspected. However, this type of radiograph presents limitations, such as the inability to reveal approximal lesions before substantial demineralization has taken place [1], as well as the risks involved with patient exposure to X-rays [19]. These limitations were clearly observed in the present study, since bitewing radiographs associated with clinical examination led to a statistically lower number of detected enamel carious lesions. Moreover, bitewing radiographs cannot be used to validate other methods for diagnosing non-cavitated lesions in contacting approximal surfaces because of its great sensibility range. Despite the challenges [1,2], early caries diagnosis is crucial for choosing the most suitable treatment option that can allow the arrest of lesion progression at initial stages [3]. On the other hand, bitewing radiographs associated with clinical examination allowed differentiation between smaller and extensive cavities within dentin (ICDAS scores 4, 5, and 6), wheres the DIFOTI-based method alone failed to differentiate between smaller and extensive carious lesions in dentin, leading to the classification of all six dentin-decayed teeth as ICDAS 4. These results suggest there might be a limitation of this device for caries detection in dentin, requiring further confirmation by clinical examination.

The present results showed the tested DIFOTI-based device was able to identify approximal carious lesions accurately, in comparison to the diagnosis set by bitewing radiographs (Table 3 and Figure 1). Furthermore, low intraoperator variability was also observed for transillumination. Thus, the first study hypothesis was accepted. These outcomes are coherent with two previously published clinical studies, which concluded that transillumination might reduce the need for bitewing radiographs for approximal carious lesions detection [9,20]. In fact, Abdelaziz et al. (2018) have reported that DIAGNOcam, the same DIFOTI device used in this study, is a reliable tool for early detection of enamel carious lesions extending up to the ADJ [10], since enamel optical properties can be altered even by slight increases in surface porosity [21]. However, the authors did not compare the accuracy of transillumination with radiographic and clinical examination, as they deemed these methods as “imperfect gold standards” [22]. Nevertheless, bitewing radiographs and clinical inspection were taken as the reference diagnostic method for the present study, regardless of their inherent limitations, as these are the most widely used techniques for caries detection in the clinical environment, and the main objective of this clinical study was to compare the feasibility of a new technology with the resources currently available for clinicians. Even so, the results of the present study should be interpreted in light of a limitation regarding the clinical exam: the lack of tooth separation by orthodontic appliances for visual inspection. This might have led to an underdetection of approximal carious lesions in the bitewing radiographs group, although the visual examination without additional procedures of tooth separation also makes the results more relevant to the reality of clinical practice of most dentists.

The transillumination method has showed higher sensitivity (ability of a test to identify a true positive result) than traditional radiographs to detect early changes in enamel [7,23]. Similarly, the results of the present study showed the high accuracy of the DIFOTI-based device to identify lesions classified as ICDAS scores 1 and 2, which solely involved enamel (Figure 1). Furthermore, the near-infrared light transillumination could be used routinely over all tooth surfaces, especially approximal ones, without any related side effects, since it does not use X-ray radiation [9]. The non-existent radiation-related risks, added to the high sensitivity, could render DIFOTI-based devices a useful tool for monitoring the outcomes of preventive treatment measures, especially in young populations [11]. Conversely, radiographs require a minimum demineralization of the dental hard tissues of 40–60% to make carious lesions clearly visible, which might lead more often to false negative results [24], i.e., low sensitivity.

There has been a concern in the literature that the application of transillumination could cause an overdetection of lesions due to its lower specificity (ability of a test to identify a true negative result) compared to radiographs [9], which was not observed in this study (Table 2). Differently from other investigations, a large number of patients (100) was evaluated in the present study, based on sample size and power analyses, which might have led to more significant and accurate results. Additionally, the statistical analyses used in this study were built upon Kuhnisch et al. (2016) [20], albeit with a slight difference: the accuracy of DIFOTI was evaluated according to each ICDAS score established *a priori* by bitewing radiographs coupled with clinical examination. Moreover, the lower sensitivity of DIFOTI- based techniques might be attributed to the lack of adequate control methods for caries diagnosis as discussed above, since bitewing radiographs and clinical inspection are used as true findings despite their limitations [11].

The accuracy of near-infrared light transillumination (NILTI) in comparison with BW was stated by a recent systematic review and meta-analysis [25]. Moreover, other studies confirmed the best performance of NILTI in comparison with other methods [26].

The time required to execute the acquisition of either DIFOTI-based or radiographic images was also evaluated and compared between each other. Not only did transillumination take less time to be performed (almost half the time necessary for bitewing radiographs using phosphor plates), but it also showed lower time variability among multiple procedures, which suggests this method might be more straightforward and standardized for clinicians, leading to the acceptance of the second study hypothesis. Nonetheless, the fact that the bitewing radiographs used in this study were acquired by phosphor plates, i.e., a hybrid manual-digital technique, should be taken into account, as this will obviously take more time compared to completely digital solutions. Therefore, DIFOTI-based devices could easily be used by dental hygienists in order to perform an initial triage of the cases that would surely require attention from the clinician for caries management, shortening the clinical time necessary for diagnostic procedures and making treatment decisions easier.

However, the high cost of DIFOTI-based devices or other near-infrared devices [27] may be considered the biggest drawback that still limits their use in everyday clinical practice, since the acquisition of the device itself and respective software, coupled with the time necessary to train personnel, could increase the overall costs of caries diagnostic procedures. Additionally, despite current published studies, including the results herein presented, showing advantages of transillumination for caries detection, the replacement of traditional techniques always takes time until a new diagnostic tool can really infiltrate clinical daily life and show itself worthy of the investment by practitioners. These limitations are especially important in the public health system context in many underdeveloped countries, where the use of standard manual radiographic techniques is still in place.

## 5. Conclusions

The transillumination device DIAGNOcam seems to provide accurate detection of approximal carious lesions in posterior teeth, even at their early stages with no dentin involvement. Additionally, the technique employed for transillumination caries diagnosis takes less time than conventional bitewing radiographs, which might lead to faster dental visits for the patients, who would not be exposed to radiation-related risks, while also making the work of dental professionals more straightforward and easier.

However, an important limitation of the present study should be taken into consideration, as the supposed carious lesions diagnosed by the tested methods were not evaluated histologically, because the patients’ teeth were not extracted after being analyzed. Therefore, the presence of carious lesions was not confirmed outside the clinical setting, which would be interesting for future studies correlating the histological, microscopic aspect of the lesions with the diagnostic accuracy of bitewing radiographs in comparison to transillumination.

## Figures and Tables

**Table 1:** Comparison between the clinical steps required by the two diagnostic techniques.

Bitewing Radiographs with Phosphor Plates	DIFOTI Images Taken with DIAGNOcam
1. Place the protection mantle over the patient;	1. Attach the occlusal tip into the handpiece and cover it with a disposable barrier;
2. Insert the photostimulable phosphor plate in the Rinn film holder;	2. Start up the DIAGNOcam software, and create or select a patient;
3. Place the film holder with the plate in the patient’s mouth;	3. Contact the light apertures to the gingiva;
4. Position correctly the cylinder from the X-ray machine;	4. Place the spacer of the occlusal probe on the neighboring tooth, and monitor the live picture;
5. Set exposure parameters and execute X-ray;	5. Tip the probe slightly, if needed;
6. Remove the protection mantle from the patient;	6. Select the tooth in the tooth diagram for which a picture will be saved;
7. Insert the phosphor plate into the scanner after removing its protective shield;	7. Press the ring switch to create a still picture and save it.
8. Acquire and record radiographs into the clinical chart.	

**Table 2:** Number of tooth surfaces identified as each ICDAS score, according to the diagnostic method used (bitewing radiographs or transillumination).

ICDAS Scores	Bitewing Radiographs + Clinical Examination	DIFOTI-Based Device	*p* Value
0	3249	3227	0.001
1	120	77	0.002
2	105	140	0.022
3	40	70	0.004
4	3	6	0.318
5	3	0	0.084
6	0	0	NA

NA: not applicable.

**Table 3:** Area under receiver operator characteristic curves (AUROC) of the transillumination technique for each ICDAS score derived from bitewing radiographs.

ICDAS Scores	ROC Area (Standard Error)	95% Confidence Interval	*p* Value
0	0.766 (0.019)	0.729–0.802	<0.0001
1	0.717 (0.029)	0.660–0.773	<0.0001
2	0.783 (0.029)	0.726–0.839	<0.0001
3	0.815 (0.044)	0.728–0.901	<0.0001

## Data Availability

All data can be requested to the corresponding author (MRA Gatto mariarosaria.gatto@unibo.it or to G.A. Pelliccioni gian.pelliccioni@unibo.it.
